# sPIF promotes myoblast differentiation and utrophin expression while inhibiting fibrosis in Duchenne muscular dystrophy via the H19/miR-675/let-7 and miR-21 pathways

**DOI:** 10.1038/s41419-019-1307-9

**Published:** 2019-01-28

**Authors:** Daria Morgoulis, Peter Berenstein, Simona Cazacu, Gila Kazimirsky, Amir Dori, Eytan R. Barnea, Chaya Brodie

**Affiliations:** 1 The Mina and Everard Goodman Faculty of Life Sciences, Bar-Ilan University, Ramat-Gan, Israel; 2 Department of Neurosurgery, Henry Ford Hospital, Detroit, MI USA; 3 Department of Neurology, Talpiot Medical Leadership Program, Chaim Sheba Medical Center, Ramat-Gan, and Sackler Faculty of Medicine, Tel Aviv University, Tel Aviv, Israel; 4 BioIncept, LLC, New York, NY 10016 USA

## Abstract

Duchenne muscular dystrophy (DMD) is a progressive, lethal, X-linked disease of skeletal and cardiac muscles caused by mutations in the dystrophin gene. Loss of dystrophin leads to muscle fiber damage and impairment of satellite cell asymmetric division, which are essential for muscle regeneration. These processes ultimately result in muscle wasting and the replacement of the degenerating muscles by fibrogenic cells, a process that leads to the generation of fibrotic tissues. Preimplantation factor (PIF) is an evolutionary conserved 15-amino acid peptide secreted by viable mammalian embryos. Synthetic PIF (sPIF) reproduces the protective/regenerative effects of the endogenous peptide in immune disorders and transplantation models. In this study, we demonstrated that sPIF treatment promoted mouse and human myoblast differentiation and inhibited the expression of collagen 1A1, collagen 1A2, and TGF-β in DMD patient-derived myoblasts. Additionally, sPIF increased the expression of utrophin, a homolog of dystrophin protein. sPIF effects were mediated via the upregulation of lncRNA H19 and miR-675 and downregulation of let-7. sPIF also inhibited the expression of miR-21, a major fibrosis regulator. The administration of sPIF in mdx mice significantly decreased serum creatine kinase and collagen I and collagen IV expression in the diaphragm, whereas it increased utrophin expression in the diaphragm, heart and quadriceps muscles. In conclusion, sPIF promoted the differentiation of DMD myoblasts, increased utrophin expression via the H19/miRNA-675/let-7 pathway, and reduced muscle fibrosis possibly via the upregulation of miR-675 and inhibition of miR-21 expression. These findings strongly support pursuing sPIF as a potential therapeutic agent for DMD. Moreover, the completion of an sPIF phase I safety trial will further promote the use of sPIF for the treatment of muscular dystrophies.

## Introduction

Duchenne muscular dystrophy (DMD) is a recessive, fatal, X-linked disease and the most common form of muscular dystrophy^1^. DMD is caused by mutations in the dystrophin gene that can be either spontaneous or inherited^2,3^. The dystrophin protein plays a critical role in the maintenance, integrity, and normal functions of muscle cells, and its loss leads to progressive muscle degeneration^4,5^. Under normal conditions, muscle damage activates quiescent muscle stem cells (satellite cells), which eventually differentiate into mature muscle cells and contribute to tissue regeneration^6^. Recent studies indicate that dystrophin is also essential for the asymmetric division of satellite cells, and the lack of functional protein interferes with the regenerative capacity of these cells^7^. Thus, muscle regeneration is impaired in DMD, resulting in the replacement of degenerating muscle fibers by fibrotic and fat tissues^1,8^.

Various therapeutic approaches have been explored for treating DMD, including promotion of muscle regeneration^9^, anti-inflammatory and anti-fibrotic agents^2,10,11^, and exon-skipping with antisense oligonucleotides^12,13^. Despite these efforts, there is currently no cure for DMD, and treatments focus mainly on relieving the symptoms and minimizing complications^14^.

Preimplantation factor (PIF) is a 15-amino acid peptide secreted by viable embryos, which through autotrophic action promotes development and protects against embryo demise due to adverse environments^15–18^. PIF promotes implantation and placental engraftment, and reduces spontaneous and Lipopolysaccharide (LPS)-induced fetal demise in an immune intact murine model^19–21^. The synthetic PIF analog (sPIF) replicates the native peptide actions and has comprehensive immune protective and regenerative properties^22–24^. sPIF targets innate immunity (macrophages/neutrophils) and affects activated T-cell proliferation and mixed lymphocyte reaction^22^ as well as decreased NK cytotoxicity^25^. Through an integrated local and systemic effect, sPIF protects against juvenile diabetes^26^, cardiovascular inflammation^27^, radiation-induced injury^28^, neuroinflammation, and neurotrauma in various animal models^29–32^. In addition, sPIF promotes ovarian allo-transplantation in transplantation models in primates and semi and allogeneic bone marrow transplantation^33,34^. Recently, sPIF was the subject of a successful University-initiated FDA Fast-Track-awarded Phase I clinical trial for autoimmune hepatitis and obtained Orphan Drug Designation (www.clinicaltrials.gov, NCT 02239562).

In this study, we demonstrated that sPIF increased muscle cell differentiation and utrophin level while inhibiting fibrogenic gene expression and identified the H19/miR-675/let-7 and miR-21 pathways as mediators of sPIF effects. Moreover, sPIF administration to mdx mice decreased creatine kinase (CK) levels and tissue fibrosis and increased the expression of utrophin, thus inducing a change in biomarkers that suggests a therapeutic effect.

## Results

### sPIF increases the differentiation of human and mouse myoblasts and decreases collagen and TGF-β expression

**Fig. 1:**
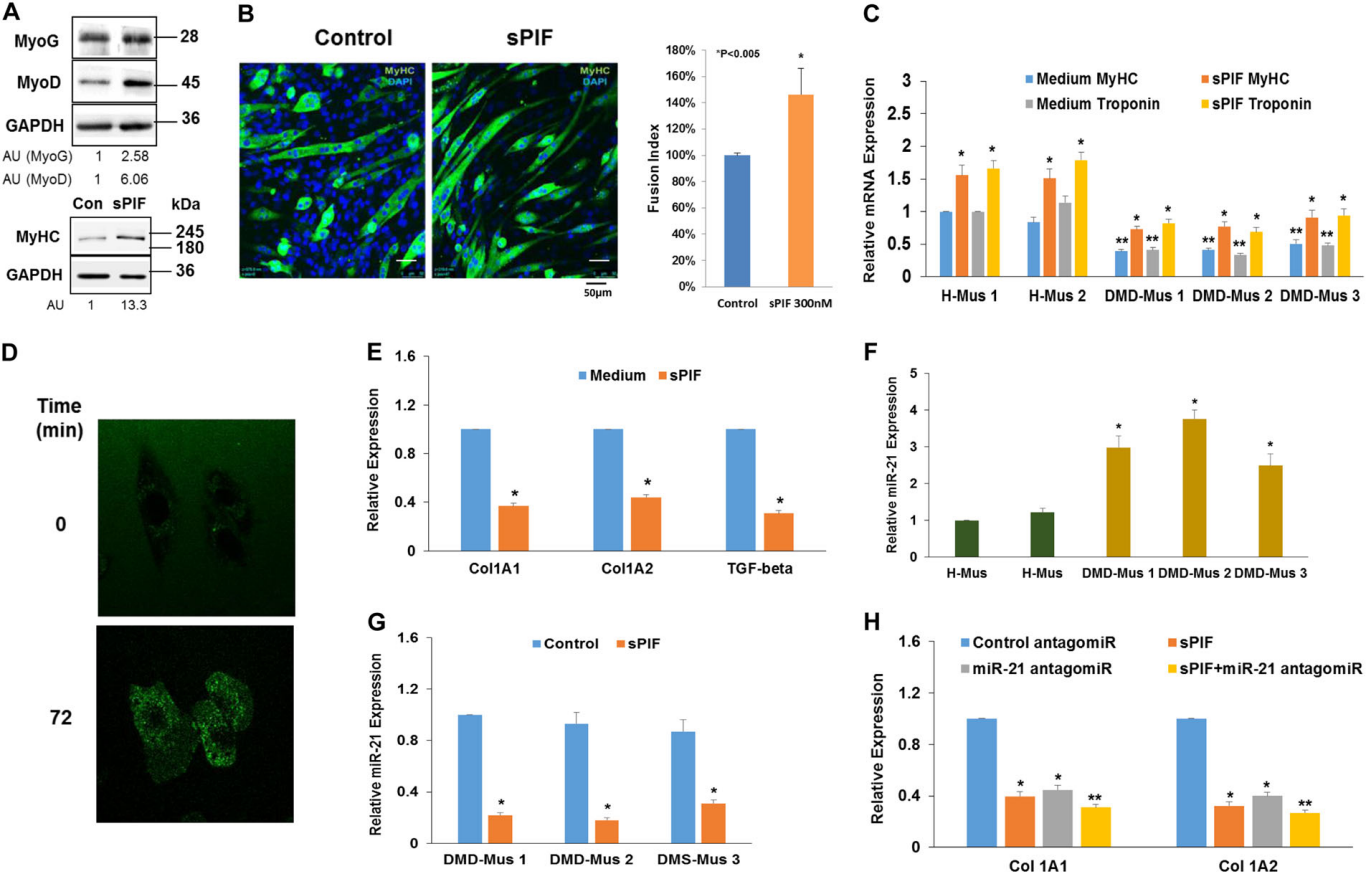
sPIF increases the differentiation of mouse and human myoblasts. C2C12 cells and human myoblasts were cultured in 2% HS and treated with sPIF (300ng/ml) for 10 days. The expression of the different myogenic proteins was determined using western blot analysis (*P < 0.001, control vs sPIF-treated cells; **P < 0.01, MyHC and troponin expression in control vs DMD muscle cells) (**a**). C2C12 cells were cultured in 2% HS and after 10 days stained with DAPI and anti-myosin heavy chain (MyHC) antibody (FITC). Cell fusion was analyzed using confocal microscopy (**b**). Human myoblasts derived from three DMD patients (DMD-mus) and two healthy donors (H-mus) were treated with sPIF (300ng/ml) and analyzed for the expression of MyHC and troponin following 10 days in fusion medium using RT-PCR (*P* < 0.001) (**c**). The internalization of sPIF-FITC into C2C12 cells was analyzed by live cell imaging using confocal microscopy. The fluorescence of the cells at the initial stage of the experiment and after 72 min is presented (**d**). The effects of sPIF (300 nM, 2 days) on the expression of Col 1A1, Col 1A2, and TGF-β in DMD myoblasts were determined using RT-PCR (*P* < 0.001) (**e**). The expression of miR-21 was determined in muscle cells obtained from healthy donors and DMD patients (**f**) and in DMD muscle cells treated with sPIF (*P < 0.001) (**g**). The effects of miR-21 antagomiR on the expression of Col 1A1 and Col 1A2 in control and sPIF-treated DMD muscle cells were determined after 3 days of treatment (*P < 0.001 for sPIF and miR-21 antagomiR vs control; **P < 0.01 for sPIF+miR-21 antagomiR vs sPIF alone) (**h**). The results are representative of three similar experiments and are presented as the means ± SE. DAPI, 4′,6-diamidino-2-phenylindole; DMD, Duchenne muscular dystrophy; HS, horse serum; RT-PCR, real-time polymerase chain reaction; sPIF, synthetic PIF analog

To study the effects of sPIF, we employed two in vitro systems: mouse C2C12 cells and human myoblasts derived from healthy donors and DMD patients. We first examined the effects of sPIF on myoblast differentiation by analyzing the expression of the myogenic factors, MyoD and myogenin, which are required for the proper differentiation of myogenic cells during the differentiation and repair of myosin heavy chain (MyHC), which is expressed in differentiated muscle cells. The fusion of myoblasts into differentiated myotubes was also determined. Myoblasts were cultured in a medium containing 2% horse serum for 10 days to allow cell differentiation. sPIF was added to the cells at the beginning of the culture and every 2 days thereafter. Treatment of C2C12 cells with sPIF increased the expression of MyoD, myogenin, and MyHC (Fig. 1a) as well as cell fusion (Fig. 1b). Similarly, sPIF increased the expression of MyHC and troponin in human myoblasts derived from both healthy donors and DMD patients (Fig. 1c). The internalization of sPIF in muscle cells was followed using Fluorescein isothiocyanate (FITC)-labeled sPIF and confocal microscopy. As presented in Fig. 1d, sPIF accumulated in the cells within 72 min of incubation and was localized in the cytoplasm.

Muscles from DMD patients and mdx mice exhibit increased tissue fibrosis that is due, partly, to the increased transdifferentiation of myoblasts into myofibroblasts. Indeed, recent studies from our laboratory demonstrated that muscle cells from DMD patients express increased fibrogenic phenotypes compared to control cells^35^. In contrast to its promoting effect on the myogenic differentiation of DMD myoblasts, sPIF inhibited the fibrogenic differentiation of these cells and decreased the expression of collagen 1A1 and 1A2 and that of TGF-β (Fig. 1e), which plays a major role in fibrogenesis. Similar results were obtained in two additional DMD myoblasts (data not shown). Another important factor that plays a central role in tissue fibrosis is miR-21^36^. We found that human muscle cells from DMD patients expressed higher levels of miR-21 compared with control muscle cells (Fig. 1f). Treatment with sPIF (300 nM) significantly decreased the expression of miR-21 in DMD muscle cells (Fig. 1g).

Silencing of miR-21 in DMD cells inhibited the expression of collagens 1A1 and 1A2 (Fig. 1h), and the treatment with sPIF induced an additional modest decrease. These effects were repeated in two additional DMD muscle cells. The results suggest that the downregulation of miR-21 expression by sPIF plays a role in its inhibitory effects on the fibrogenic differentiation of these cells and that additional pathways may be also involved.

### sPIF increases myoblast differentiation via the induction of lncRNA H19 and miR-675

**Fig. 2:**
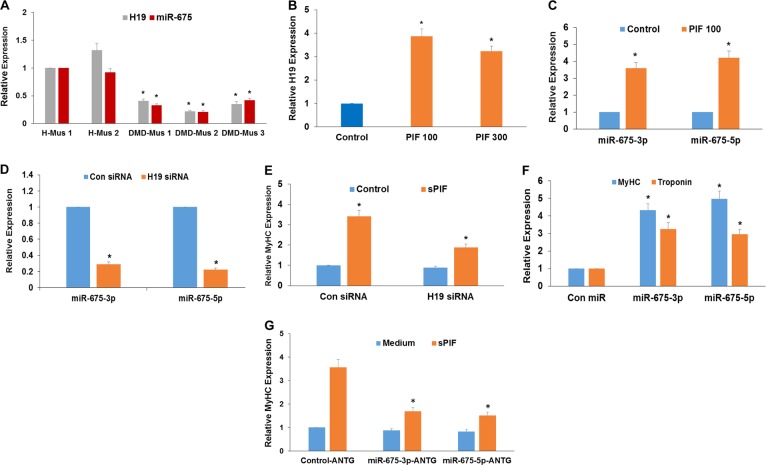
sPIF increases the differentiation of DMD myoblasts via the upregulation of the H19/miR-675 pathway. The expression of H19 and miR-675 was analyzed in cultured muscle cells derived from healthy donors and DMD patients using RT-PCR (*P < 0.001) (**a**). sPIF induced the upregulation of both H19 (**b**) and miR-675–3p and -5p expression (**c**) as determined by RT-PCR (*P < 0.001). DMD myoblasts were transfected with a control or H19 siRNA. The expression of miR-675 (**d**) and the induction of MyHC by sPIF were determined by RT-PCR (*P < 0.001 for untreated vs/sPIF-treated cells and control vs H19 siRNA cells treated with sPIF) (**e**). DMD myoblasts were transfected with miR-675–3p or miR-675–5p mimics, and the expression of MyHC and troponin was determined 7 days later (*P < 0.001 for MyHC and troponin expression of con siRNA vs miR-675-overexpressing cells) (**f**). The effect of sPIF on MyHC expression was determined in DMD myoblasts transfected with a control or miR-675 antagomiRs (*P < 0.001 for sPIF-treated cells expressing miR-675 antagomiRs vs cells expressing a control antagomiR) (**g**). The results are representative of at least three similar experiments and presented as the means  ± SE. DMD, Duchenne muscular dystrophy; MyHC, myosin heavy chain; RT-PCR, real-time polymerase chain reaction; sPIF, synthetic PIF analog

To further analyze the mechanisms involved in the effects of sPIF on muscle differentiation, we focused on the H19/miR-675 pathway. Long non-coding RNA (lncRNA) H19 plays an essential role in muscle differentiation and regeneration, and its biological functions are mediated by miR-675–3p and miR-675–5p, which are encoded by exon 1 of H19^37^. Myoblasts derived from DMD patients expressed significantly reduced levels of H19 and miR-675 compared to myoblasts derived from healthy donors (Fig. 2a). The treatment of DMD myoblasts with sPIF significantly upregulated the expression of both H19 (Fig. 2b) and miR-675 (Fig. 2c) in these cells, and these effects were observed in two additional DMD muscle cells (sPIF 100 nM; DMD-Mus2–H19: 398.4 ± 4.2%; miR-675: 428.96 ± 5.11%; DMD-Mus3–H19: 312 ± 39.6; miR-675: 287 ± 23.1% compared to 100% in control untreated cells).

To demonstrate that the induction of miR-675 played a role in sPIF-induced myoblast differentiation we performed two experiments. First, we transfected DMD myoblasts with an siRNA duplex that targets H19 and showed that the silencing of H19 decreased miR-675 expression in myoblasts (Fig. 2d) and their differentiation by sPIF (Fig. 2e). We next transfected the DMD myoblasts with miR-675–3p and miR-675–5p mimics and found that the expression of MyHC and troponin was significantly increased after 5 days of treatment (Fig. 2f). We also examined the effects of sPIF on the differentiation of myoblasts transfected with miR-675 antagomiRs and found that its effects were markedly abrogated, whereas, no significant decrease in the effects of sPIF was observed in myoblasts transfected with a control antagomiR (Fig. 2g). These results indicate that the induction of H19 and the subsequent upregulation of miR-675 mediated, at least partly, some of the increased differentiation of the human myoblasts by sPIF. These results exhibited similar patterns in additional DMD muscle cells (data not shown).

### sPIF increases utrophin expression via the H19/miR-675/let-7 pathway

**Fig. 3:**
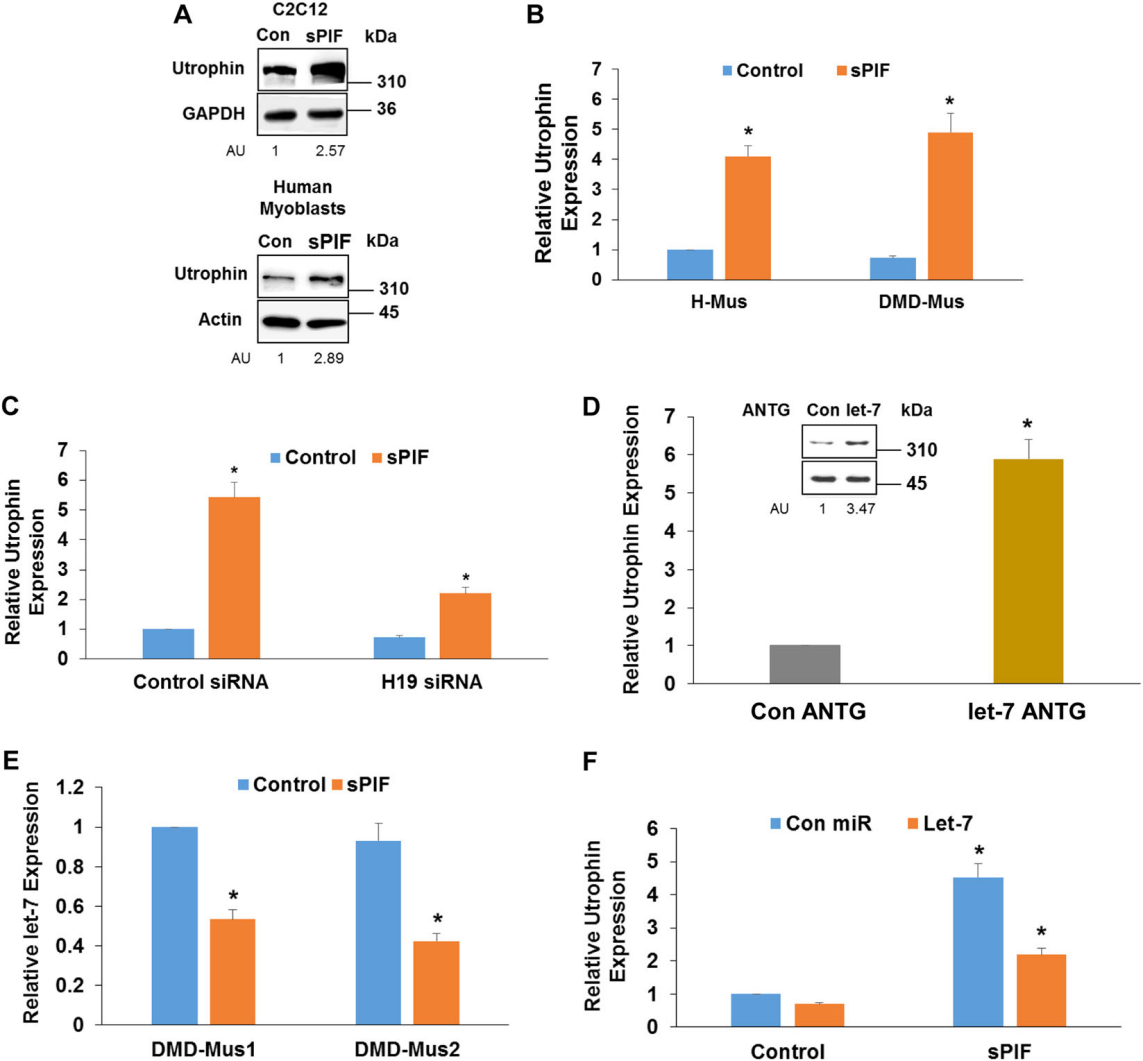
sPIF upregulates utrophin expression via the H19/Let-7 pathway. C2C12 and human myoblasts were treated with sPIF for 10 days and utrophin expression was determined by western blot analysis (**a**). Utrophin expression was also analyzed in sPIF-treated DMD myoblasts as determined by RT-PCR (**b**). DMD myoblasts were silenced for H19, and the effects of sPIF on utrophin expression were determined following 5 days of treatment (**c**). DMD myoblasts were treated with a control or let-7 antagomiR, and utrophin expression was analyzed 3days later using RT-PCR and western blot analysis (**d**). The expression of let-7 was determined in DMD myoblasts following sPIF treatment for 3 days by RT-PCR (**e**). DMD myoblasts were transfected with a control or let-7 mimic and then treated with sPIF for 5 days. Utrophin expression was determined by RT-PCR (**f**). The results are representative of at least three similar experiments and presented as the means ± SE. *P < 0.001. DMD, Duchenne muscular dystrophy; RT-PCR, real-time polymerase chain reaction; sPIF, synthetic PIF analog

Utrophin is a functional and structural autosomal paralog of dystrophin. These proteins share binding partners, and upregulating utrophin expression is considered a potential therapeutic approach in DMD. In addition to its effect on myoblast differentiation, sPIF treatment also increased the expression of utrophin in C2C12 (Fig. 3a) and human muscle cells (Fig. 3a, b). This effect of utrophin was observed in additional muscle cultures from healthy and DMD donors (sPIF treated—H-Mus2: 452.3 ± 40.2%; DMD-Mus2: 532.8 ± 48.2%; DMD-Mus3: 387.1 ± 30.6% compared to 100% in control untreated cells). The induction of utrophin in human myoblasts was partly dependent on the increased expression of H19 since silencing of this lncRNA abrogated the upregulation of utrophin expression (Fig. 3c). H19 acts as a sponge of let-7^38^, which targets the 3’-UTR of utrophin^39,40^, suggesting that the upregulation of H19 may interfere with the ability of let-7 to target utrophin. We, therefore, examined the effects of let-7 silencing on utrophin expression in DMD myoblasts and found an increased utrophin expression in the let-7-silenced myoblasts (Fig. 3d). Since sPIF was recently reported to downregulate let-7 expression in neural and lymphoid cells by decreasing its biogenesis, we examined the effects of sPIF on let-7 expression also in muscle cells^30^. Treatment of human myoblasts with sPIF decreased the expression of let-7 (Fig. 3e), and the overexpression of let-7 abrogated the upregulation of utrophin by sPIF (Fig. 3f). These results suggest that sPIF may increase utrophin expression by decreasing let-7 expression and by inhibiting the ability of let-7 to target utrophin via the upregulation of H19.

### sPIF decreases CK levels and muscle tissue fibrosis in mdx mice

**Fig. 4:**
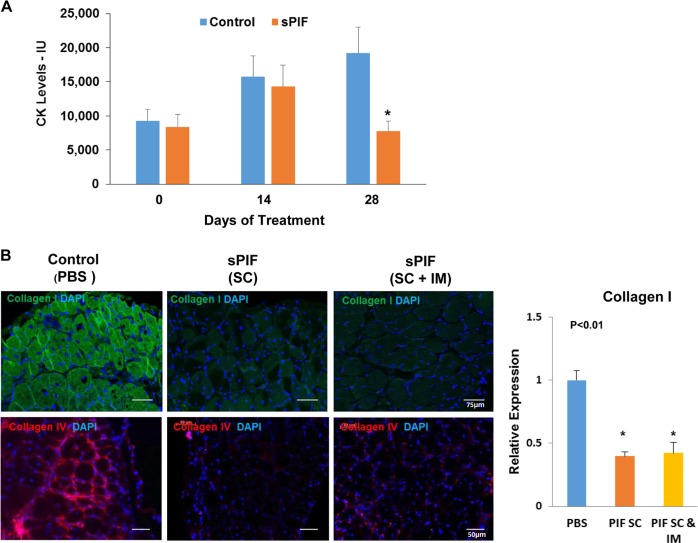
sPIF decreased CK levels and tissue fibrosis in mdx mice. Mice (6 weeks; *n* = 8 per group) were injected with PBS (controls) or sPIF (0.75 mg/kg twice daily for 2 weeks) either SC or once SC and once to the QC muscle. CK levels were analyzed at the end of the treatment and following additional 2 weeks (*P < 0.01) (**a**). Diaphragm muscles from the treated mice were removed and stained for anti-collagen I and collagen IV (**b**). The levels of collagen I and collagen IV were analyzed for fluorescence signal intensity using CellProfiler software tool and presented as a graph (*P < 0.01). CK, creatine kinase; PBS, phosphate-buffered saline; QC, quadriceps; SC, subcutaneously; sPIF, synthetic PIF analog

We further studied the therapeutic effects of sPIF on mdx mice that represent a pre-clinical model of DMD and have a premature stop codon mutation on exon 23 of the dystrophin gene, which results in a lack of the mature protein. sPIF (0.75 mg/kg) was administered twice a day subcutaneously (SC) or SC and intramuscularly to the quadriceps muscles (QC) for 2 weeks. Mice were sacrificed at the end of the treatment and 2 weeks thereafter. We found that sPIF treatment significantly decreased serum CK levels when examined 4 weeks post administration (Fig. 4a), suggesting that sPIF exerted a therapeutic impact in this disease model.

Muscle fibrosis is one of the hallmark processes in DMD, resulting from muscle degeneration and correlating with patient functional deterioration^41,42^. In mdx mice, fibrosis is mainly observed in the diaphragm and heart^43,44^. We therefore analyzed the effects of sPIF on fibrosis in these tissues. Mice treated with sPIF for 2 weeks were sacrificed 2 weeks later, and the diaphragm tissues were analyzed for collagen I and IV expression using immunofluorescence staining. The expression of collagen I and IV in the diaphragm of sPIF-treated mice was significantly lower compared with that in the tissues of control mice (Fig. 4b). In contrast, sPIF did not induce a significant decrease in collagen I or IV expression in cardiac tissues (data not shown).

### sPIF increases utrophin expression in muscle tissues

**Fig. 5:**
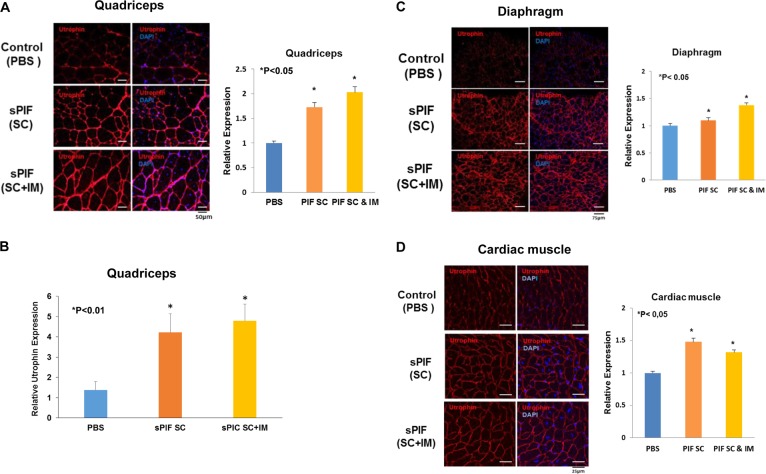
sPIF increases the expression of utrophin in mdx mice. Mice were injected with PBS or sPIF as described in Fig. 4. Following 2 additional weeks, the quadriceps (**a**), diaphragm (**c**), and cardiac (**d**) muscle tissues were immunostained with an anti-utrophin antibody (red) and the sections visualized by confocal microscopy. The levels of utrophin were analyzed for fluorescence signal intensity using CellProfiler software tool and presented as a graph. Tissue sections of the quadriceps muscle were also analyzed for the expression of utrophin using RT-PCR (**b**). The results are representative of at least three similar experiments and presented as the means ± SE (*P < 0.001). PBS, phosphate-buffered saline; RT-PCR, real-time polymerase chain reaction; sPIF, synthetic PIF analog

Recent studies have reported an inverse correlation between fibrosis and utrophin expression in both patient biopsies and mdx mice^44^. We found that sPIF increased utrophin in cultured cells and therefore examined the effects of sPIF also on utrophin expression in mdx muscle. sPIF treatment significantly increased utrophin expression in the QC (Fig. 5a, b), diaphragm (Fig. 5c), and cardiac (Fig. 5d) muscle tissues of mdx mice.

## Discussion

DMD is an X-linked muscle degenerative disease that currently has no cure. New therapeutic strategies being pursued for the treatment of this disease include steroids^45^, cell-based therapies^46^, and increasing functional dystrophin expression^47^.

In this study, we focused on sPIF as a potential therapeutic agent for the treatment of DMD and on analyzing the molecular mechanism underlying its effects using both mouse and human in vitro models and mdx mice.

The immune regulatory and regenerative actions of sPIF have been recently reported^22,48^. sPIF promotes embryo differentiation to the blastocyst stage through a targeted action and protects against oxidative stress and protein misfolding^15–18^. The trophic and protective actions of sPIF were also confirmed by demonstrating its effects on reducing inflammation and promoting neural repair and re-myelination in the spinal cord and brain^29,32,48^. Similarly, in a newborn hypoxic ischemic model, sPIF treatment was able to restore the size of the injured cortex^30,31^. sPIF mostly regulates activated immune response without leading to immune suppression and preserves anti-pathogen action^22^. Thus, sPIF may limit damage and inflammation and promote tissue regeneration^28,30,34^.

sPIF increased the differentiation of both C2C12 cells and human myoblasts derived from healthy donors and DMD patients, whereas it inhibited the fibrogenic phenotypes of DMD myoblasts. Muscle cells derived from DMD patients exhibited decreased expression of lncRNA H19 and miR-675 compared to healthy donor-derived cells. H19 has been identified as an important lncRNA in the function of skeletal muscle differentiation by increasing the expression of miR-675, which in turn inhibits the TGF-β pathway^37^. sPIF increased the expression of H19, miR-675–3p, and miR-675–5p in DMD myoblasts and the differentiation of these cells in an miR-675-dependent manner. This is the first study that demonstrates the effect of sPIF on DMD myoblast differentiation and identifies the H19/miR-675 pathway as a mediator of this effect.

In addition to the in vitro effects, sPIF also exerted a therapeutic impact in mdx mice, which are used as a DMD animal model^49^. Treatment with sPIF significantly decreased the serum CK levels in the treated mice, which reflects the decreased muscle degeneration and tissue damage.

Tissue fibrosis is one of the characteristic pathological hallmarks of DMD and represents a secondary process to the degeneration of muscle tissues^41,50^. Muscle fibrosis is directly correlated with disease progression and poor prognosis^51^. Tissue fibroblasts are considered the main mediators of this process; however, recent studies have reported that DMD myoblasts can also contribute to muscle fibrogenesis^50^. We found that sPIF decreased the fibrosis levels in the diaphragm of the mdx mice as indicated by the decreased immunofluorescence staining of collagen I.

sPIF inhibited the expression of the fibrogenic phenotype and TGF-β expression in DMD muscle cells. sPIF also decreased the expression of miR-21, which was higher in DMD muscle cells compared with control myoblasts. miR-21 plays a major role in the fibrosis of various tissues, including liver, renal, and cardiac muscle, and in age-associated muscular dystrophy^36,52–54^. Thus, the upregulation of miR-675 and the decreased miR-21 expression, which target the TGF-β pathway and collagen expression, respectively, may mediate the inhibitory effect of sPIF on tissue fibrosis.

Similar to its effects in cultured myoblasts, sPIF also induced the expression of utrophin in the QC, diaphragm, and cardiac tissues of mdx mice. Utrophin is a homolog of dystrophin protein, which is mainly expressed during the embryonic development of skeletal muscles and replaced by dystrophin postnatally^55^. Upregulation of utrophin levels is considered a potential therapeutic approach for the treatment of DMD since this protein can substitute for some of the dystrophin functions in the muscles of DMD patients^56^. Recent studies have reported that utrophin is directly targeted by let-7 via binding to its 3‘-UTR. Our results demonstrate that sPIF downregulates let-7 expression thereby providing a potential mechanism for the upregulation of utrophin by sPIF. Indeed, sPIF was reported to downregulate let-7 expression also in neuronal and lymphoid cells by inhibiting its biogenesis^30^. Another mechanism by which sPIF increases utrophin levels may be via the upregulation of H19 expression. H19 acts as a sponge of let-7 and therefore can interfere with the targeting of utrophin by let-7. Interestingly, the expression of utrophin has been reported to be inversely correlated with the level of fibrosis in DMD muscle^44^, which can further contribute to the upregulation of utrophin secondary to the decreased fibrosis induced by sPIF.

**Fig. 6:**
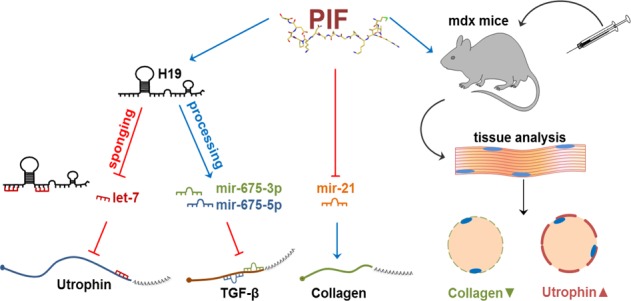
A diagram summarizing the various molecular and cellular pathways associated with sPIF effects in DMD. The different in vitro and in vivo effects of sPIF and its various pathways and mediators are depicted in this diagram

The results of this study demonstrate that sPIF promotes muscle differentiation in vitro and exerts a therapeutic impact in mdx mice by decreasing the levels of fibrosis and inducing utrophin expression. The effects of sPIF were at least partially mediated via the upregulation of the H19/miR-675 pathway and downregulation of let-7 and miR-21 (Fig. 6). Additional factors, including inflammatory cytokines and TGF-β, are also likely to play a role in sPIF effects and are currently being explored. Phase I clinical trial has been now completed for sPIF in autoimmune hepatitis. Based on this and our current results, we propose that sPIF represents a potential therapeutic agent for the treatment of DMD.

## Materials and methods

### Materials

The following antibodies were employed: myosin heavy chain (sc-376157), myoD (sc-760), Pax-7 (sc-81975), β-actin HRP-conjugated antibody (sc-47778HRP), and GAPDH (sc-26778) from Santa Cruz Biotechnology (Dallas, TX, USA); collagen I (Ab21286) and collagen IV (ab19808) from Abcam, Cambridge, UK; utrophin (NCL-DRP2) from Leica Biosystems Inc. (Buffalo Grove, IL, USA); donkey anti-rabbit Cy-3-conjugated antibody (#711–166–152) and donkey anti-mouse Cy-3-conjugated antibody (#715–165–151) from Jackson ImmunoResearch Laboratories Inc. (West Grove, PA, USA); goat anti-mouse Alexa488-conjugated antibody (#A-11029) from Molecular Probes Inc., Carlsbad, CA, USA; and 4′,6-diamidino-2-phenylindole (DAPI) (D1306) from ThermoFischer Scientific (Oregon City, OR, USA).

### Cultures of human myoblasts and C2C12 cells

Myoblasts from healthy male donors (H-Mus) and DMD patients (DMD-Mus) were obtained from ScienCell (Carlsbad, CA, USA) and from Creative Bioarray and DV Biologics (Costa Mesa, CA, USA), as previously reported^35^.

C2C12 myoblasts were maintained at subconfluent densities in Dulbecco's modified Eagle's medium (DMEM) supplemented with 10% fetal bovine serum (Invitrogen, Paisley, UK). Myogenic differentiation was induced by changing subconfluent cells to DMEM containing 2% heat-inactivated horse serum as recently reported^35^.

### Transfection of miRNA mimics, antagomiRs, and shRNAs

RNA duplexes corresponding to miR-675 and miR-21 mimics were obtained from Sigma (St. Louis, MO, USA). Transfection of the cells with the miRNA duplexes was carried out with siIMPORTER (Millipore) according to the manufacturer’s instructions and as previously reported^57^. H19 shRNA was obtained from Dharmacon, and miR-675 and miR-21 antagomiRs were obtained from SBI.

### Western blot analysis

Cell lysates (30 μg protein) were analyzed for the expression of specific markers as previously described^35^.

### Fusion analysis

C2C12 cells were plated in DMEM supplemented with 10% fetal bovine serum in a 35-mm imaging dish with an ibidi Polymer Coverslip Bottom at high density (80%) and allowed to proliferate for 48h. The medium was then replaced with a differentiation medium containing 2% horse serum, and the cells were cultured for an additional 10 days. sPIF (300 nM) was added to the culture medium at the beginning of the differentiation phase and every 48h thereafter.

Fusion index was quantified as the ratio of DAPI-stained nuclei in MyHC-positive multinucleated cells with ≥ 3 nuclei to the total number of stained nuclei. Analysis was performed using ImageJ software of 49 fields in each of the two independent culture dishes per group as previously described^35^.

### Real-time quantitative PCR analysis–

Total RNA was isolated using QIAzol reagent (Qiagen, CA, USA) according to the manufacturer's protocol. Half microgram of RNA was employed to synthesize cDNA by Thermoscript (Invitrogen) with oligo dT primers. mRNAs were analyzed by the TaqMan assay kits for troponin T (Human TNNT3 Fast skeletal muscle); myosin heavy chain type 2a fast–MyH2 (human: MyH2 Hs00952980_m1), and utrophin (human: Hs01125975_m1; mouse: Mn01168866_m1) from ThermoFisher Scientific. S12 mRNA was used as an internal control. Expression of miR-21, miR-875, and H19 was determined by the TaqMan stem-loop real-time polymerase chain reaction method. Primers and probes for TaqMan miRNA assay and the endogenous control RNU6B were purchased from System Biosciences.

### Animal studies

All animal experiments were performed in accordance with the guidelines of the Israel Board for Animal Experiments and in compliance with The Israel Animal Welfare Act and Ethics Committee. The mdx mice (6-week-old males) were housed in cages under conditions of constant photoperiod (12/12h light/dark) with free access to food and water. Mice were treated with sPIF, 0.75 mg/kg/10 μl, twice a week for 2 weeks either SC or by a combination of SC and intramuscular injections into the QC muscles. The control mice were injected with saline. The diaphragm, heart, and QC muscles were collected immediately after sPIF administration and for two additional weeks thereafter and were processed for histological and immunofluorescence analyses.

### Preparation of sections and immunofluorescence

Muscle tissues from the control and treated mice were collected and frozen in isopentane cooled with liquid nitrogen. Cryosections were prepared, fixed, and blocked with 0.2% (w/v) gelatin and 0.2% (v/v) Tween20 (Sigma Aldrich; P1379) in phosphate-buffered saline and immunostained with anti-utrophin antibody (1:50) alone or double-immunostained with collagen type I (1:100) or collagen type IV (1:50) antibodies. Cell nuclei were stained with DAPI (1:1000). As secondary antibodies, goat anti-mouse IgG antibodies with Alexa Fluor dye were used for collagen type I detection and Cy3 anti-goat for utrophin detection. Microscopic observations and image acquisition were performed with Leica SP8 Confocal and Zeiss Axioimager fluorescent microscopes and analyzed using CellProfiler software as previously reported^35^. Images from 8–12 different fields were acquired and analyzed for each mice.

### Statistical analysis

Quantitative measurements are presented as the mean values ± SE and visualized in bar charts. Data were analyzed using ANOVA or Student's *t* test, with correction for datasets with unequal variances.

## References

[1] Emery AE (2002). The muscular dystrophies. Lancet.

[2] Bushby K (2010). Diagnosis and management of Duchenne muscular dystrophy, part 1: diagnosis, and pharmacological and psychosocial management. Lancet Neurol..

[3] Bushby KM (1993). Variability in clinical, genetic and protein abnormalities in manifesting carriers of Duchenne and Becker muscular dystrophy. Neuromuscul. Disord..

[4] Blake DJ, Weir A, Newey SE, Davies KE (2002). Function and genetics of dystrophin and dystrophin-related proteins in muscle. Physiol. Rev..

[5] Keefe AC, Kardon G (2015). A new role for dystrophin in muscle stem cells. Nat. Med..

[6] Bentzinger, C. F., Wang, Y. X. & Rudnicki, M. A. Building muscle: molecular regulation of myogenesis. *Cold Spring Harb. Perspect. Biol*. **4**, 1–16 (2012). 10.1101/cshperspect.a008342 PMC3281568 22300977

[7] Dumont NA (2015). Dystrophin expression in muscle stem cells regulates their polarity and asymmetric division. Nat. Med..

[8] Uezumi A (2011). Fibrosis and adipogenesis originate from a common mesenchymal progenitor in skeletal muscle. J. Cell. Sci..

[9] Bogdanovich S (2002). Functional improvement of dystrophic muscle by myostatin blockade. Nature.

[10] Heier CR (2013). VBP15, a novel anti-inflammatory and membrane-stabilizer, improves muscular dystrophy without side effects. EMBO Mol. Med..

[11] Huebner KD, Jassal DS, Halevy O, Pines M, Anderson JE (2008). Functional resolution of fibrosis in mdx mouse dystrophic heart and skeletal muscle by halofuginone. Am. J. Physiol. Heart Circ. Physiol..

[12] Aoki Y (2012). Bodywide skipping of exons 45–55 in dystrophic mdx52 mice by systemic antisense delivery. Proc. Natl. Acad. Sci. USA..

[13] Aartsma-Rus A (2017). Development of exon skipping therapies for Duchenne muscular dystrophy: A critical review and a perspective on the outstanding issues. Nucleic Acid. Ther..

[14] Griggs RC (2016). Efficacy and safety of deflazacort vs prednisone and placebo for Duchenne muscular dystrophy. Neurology.

[15] Stamatkin CW (2011). PreImplantation Factor (PIF) correlates with early mammalian embryo development-bovine and murine models. Reprod. Biol. Endocrinol..

[16] Stamatkin CW (2011). Preimplantation factor negates embryo toxicity and promotes embryo development in culture. Reprod. Biomed. Online.

[17] Goodale LF (2017). PreImplantation factor (PIF) protects cultured embryos against oxidative stress: relevance for recurrent pregnancy loss (RPL) therapy. Oncotarget.

[18] Barnea ER (2014). Insight into PreImplantation Factor (PIF*) mechanism for embryo protection and development: Target oxidative stress and protein misfolding (PDI and HSP) through essential RIPK binding site. PLoS One.

[19] Simone NDi (2017). Synthetic PreImplantation Factor (PIF) prevents fetal loss by modulating LPS induced inflammatory response. PLoS One.

[20] Moindjie H (2016). Preimplantation factor is an anti-apoptotic effector in human trophoblasts involving p53 signaling pathway. Cell Death Dis..

[21] Paidas MJ (2010). A genomic and proteomic investigation of the impact of preimplantation factor on human decidual cells. Am. J. Obstet. Gynecol..

[22] Barnea ER (2012). PreImplantation Factor (PIF) orchestrates systemic antiinflammatory response by immune cells: effect on peripheral blood mononuclear cells. Am. J. Obstet. Gynecol..

[23] Barnea ER (2015). PIF direct immune regulation: Blocks mitogen-activated PBMCs proliferation, promotes TH2/TH1 bias, independent of Ca2+. Immunobiology.

[24] Barnea ER (2016). PreImplantation factor (PIF) regulates systemic immunity and targets protective regulatory and cytoskeleton proteins. Immunobiology.

[25] Roussev RG (2013). Preimplantation factor inhibits circulating natural killer cell cytotoxicity and reduces CD69 expression: Implications for recurrent pregnancy loss therapy. Reprod. Biomed. Online.

[26] Weiss L (2011). Preimplantation factor (PIF) analog prevents type I diabetes mellitus (TIDM) development by preserving pancreatic function in NOD mice. Endocrine.

[27] Chen YC (2016). Preimplantation factor prevents atherosclerosis via its immunomodulatory effects without affecting serum lipids. Thromb. Haemost..

[28] Shainer R (2016). PreImplantation factor (PIF) therapy provides comprehensive protection against radiation induced pathologies. Oncotarget.

[29] Weiss L (2012). Preimplantation Factor (PIF*) reverses neuroinflammation while promoting neural repair in EAE model. J. Neurol. Sci..

[30] Mueller M (2014). PreImplantation factor promotes neuroprotection by targeting microRNA let-7. Proc. Natl. Acad. Sci. USA.

[31] Mueller M (2015). PreImplantation Factor bolsters neuroprotection via modulating Protein Kinase A and Protein Kinase C signaling. Cell Death Differ..

[32] Migliara G (2017). PIF* promotes brain re-myelination locally while regulating systemic inflammation-clinically relevant multiple sclerosis M. smegmatis model. Oncotarget.

[33] Azar Y (2013). PreImplantation Factor reduces graft-versus-host disease by regulating immune response and lowering oxidative stress (Murine Model). Biol. Blood. Marrow Transplant..

[34] Feichtinger M, Barnea ER, Nyachieo A, Brännström M, Kim SS (2017). Allogeneic ovarian transplantation using immunomodulator preimplantation factor (PIF) as monotherapy restored ovarian function in olive baboon. J. Assist. Reprod. Genet..

[35] Bier A (2018). Placenta-derived mesenchymal stromal cells and their exosomes exert therapeutic effects in Duchenne muscular dystrophy. Biomaterials.

[36] Huang Y, He Y, Li J (2015). MicroRNA-21: A central regulator of fibrotic diseases via various targets. Curr. Pharm. Des..

[37] Dey BK, Pfeifer K, Dutta A (2014). The H19 long noncoding RNA gives rise to microRNAs miR-675-3p and miR-675-5p to promote skeletal muscle differentiation and regeneration. Genes Dev..

[38] Kallen AN (2013). The imprinted H19 lncRNA antagonizes Let-7 microRNAs. Mol. Cell.

[39] Basu U (2011). Translational regulation of utrophin by miRNAs. PLoS One.

[40] Mishra MK, Loro E, Sengupta K, Wilton SD, Khurana TS (2017). Functional improvement of dystrophic muscle by repression of utrophin: Let-7c interaction. PLoS One.

[41] Mann CJ (2011). Aberrant repair and fibrosis development in skeletal muscle. Skelet. Muscle.

[42] Li Y (2004). Transforming growth factor-beta1 induces the differentiation of myogenic cells into fibrotic cells in injured skeletal muscle: a key event in muscle fibrogenesis. Am. J. Pathol..

[43] Barbin IC (2016). Diaphragm degeneration and cardiac structure in *mdx* mouse: potential clinical implications for Duchenne muscular dystrophy. J. Anat..

[44] Levi O, Genin O, Angelini C, Halevy O, Pines M (2015). Inhibition of muscle fibrosis results in increases in both utrophin levels and the number of revertant myofibers in Duchenne muscular dystrophy. Oncotarget.

[45] Tidball JG, Wehling-Henricks M (2004). Evolving therapeutic strategies for Duchenne muscular dystrophy: targeting downstream events. Pediatr. Res..

[46] Farini A, Razini P, Erratico S, Torrente Y, Meregalli M (2009). Cell based therapy for duchenne muscular dystrophy. J. Cell. Physiol..

[47] Hoffman EP (2011). Restoring dystrophin expression in Duchenne muscular dystrophy muscle. Am. J. Pathol..

[48] Barnea ER (2015). Immune regulatory and neuroprotective properties of preimplantation factor: From newborn to adult. Pharmacol. Ther..

[49] Grounds MD, Radley HG, Lynch GS, Nagaraju K, De Luca A (2008). Towards developing standard operating procedures for pre-clinical testing in the mdx mouse model of Duchenne muscular dystrophy. Neurobiol. Dis..

[50] Wynn T (2008). Cellular and molecular mechanisms of fibrosis. J. Pathol..

[51] Desguerre I (2009). Endomysial fibrosis in Duchenne muscular dystrophy: a marker of poor outcome associated with macrophage alternative activation. J. Neuropathol. Exp. Neurol..

[52] Ardite E (2012). PAI-1-regulated miR-21 defines a novel age-associated fibrogenic pathway in muscular dystrophy. J. Cell. Biol..

[53] Reddy, S. et al. miR-21 is associated with fibrosis and right ventricular failure. *JCI Insight* **2**, e91625 (2017). 10.1172/jci.insight.91625 PMC5414555 28469078

[54] Liu Y (2016). TGF-β1 promotes scar fibroblasts proliferation and transdifferentiation via up-regulating MicroRNA-21. Sci. Rep..

[55] Miura P, Jasmin BJ (2003). Utrophin upregulation for treating Duchenne or Becker muscular dystrophy: how close are we?. Trends Mol. Med..

[56] van Deutekom JC, van Ommen GJ (2003). Advances in Duchenne muscular dystrophy gene therapy. Nat. Rev. Genet..

[57] Bier A (2013). MicroRNA-137 is downregulated in glioblastoma and inhibits the stemness of glioma stem cells by targeting RTVP-1. Oncotarget.

